# Healthcare resource utilization and costs among patients with rheumatoid arthritis on biologic therapies in Taiwan: A 1-year mirror-image study using a national claims database

**DOI:** 10.1371/journal.pone.0200758

**Published:** 2018-07-18

**Authors:** Kuan-Chen Chen, Chu-Hua Wu, Chao-Hsiun Tang, Kuo-Cherh Huang

**Affiliations:** 1 School of Health Care Administration, College of Management, Taipei Medical University, Taipei, Taiwan; 2 Department of Health Care Management, National Taipei University of Nursing and Health Sciences, Taipei, Taiwan; Charles P. Darby Children's Research Institute, UNITED STATES

## Abstract

**Objectives:**

This nationwide population-based study aimed at evaluating healthcare resource utilization and direct medical costs among rheumatoid arthritis (RA) patients receiving biologic therapies in Taiwan.

**Design and setting:**

A retrospective cohort of 2,425 RA patients who had received first-line tumor necrosis factor (TNF)-α antagonist treatment for at least 6 months (the baseline period) between 2007 and 2011 was identified from the National Health Insurance Research Database in Taiwan.

**Outcome measures:**

Healthcare resource utilization and direct medical costs of those patients were analyzed and compared 1 year before the index date and during the 1-year follow-up.

**Results:**

Analytical results demonstrated that 87.7% of RA patients received the same TNF-α antagonist during the 1-year follow-up, 2.4% of the patients switched to another TNF-α antagonist after the baseline period, 7.1% of the study cohort received a second-line biologic agent, while the remaining patients discontinued use of any TNF-α antagonist. Compared to 1 year before the index date, there were significant reductions in emergency room visits and hospitalization days for RA patients treated with the same TNF-α antagonist during the 1-year follow-up. However, there was an increase of outpatient visits among those patients. For those RA patients who switched to another TNF-α antagonist or received a second-line biologic agent, they consumed more healthcare resources. Furthermore, the corresponding medication costs went up markedly during the 1-year follow-up, but nearly all total direct medical costs (biologics excluded) were significantly reduced across the study cohort. Lastly, male patients incurred slightly higher medical costs than their counterparts, albeit in a statistically insignificant fashion.

**Conclusions:**

This investigation revealed that RA patients treated with biologics utilized fewer emergency room visits and shorter hospitalization days, but incurred higher costs. In summary, this study provides meaningful information on healthcare resource utilization and medical costs of RA patients for healthcare providers and policymakers.

## Introduction

Rheumatoid arthritis (RA) is a chronic inflammatory disease caused by the immune system attacking joints. RA can lead to chronic arthritis and inflammation of other organs such as heart and lung, and is one of the leading causes of disability worldwide. According to the World Health Organization (WHO), global prevalence of RA varies in the range of 0.3% to 1%, and is more common in women and in developed countries [1]. Although the mortality of RA patients is comparatively low, the economic burden is substantial due to treatment costs and productivity losses, and has been well assessed in Western nations [2–6]. In a nationally representative panel survey in the US in 2008, the adjusted average annual healthcare expenditures of the RA cohort were $13,012 compared with $4,950 of a non-RA control cohort. The higher expenditures of the RA patients were primarily driven by higher costs for drug treatment [6].

Novel therapy of RA patients with biologic agents has been considerably prescribed in recent years. Currently a number of biologics have been approved and have demonstrated to be effective in reducing RA symptoms, ameliorating disease progression, and improving health-related quality of life [7,8]. Nonetheless, as the number of patients treated with biologics has been increasing steadily over the past few decades, employers, insurers, and policymakers are growing concerned about the rising costs of biologic therapies since they are largely expensive in terms of costs per dose.

There are a number of biologics currently available for the treatment of RA, including Adalimumab, Etanercept, Rituximab, among others. As the availability of new treatments for RA increases, it is important for healthcare providers and policymakers to be aware of healthcare resource utilization and associated medical costs as RA exerts a significant burden on both patients and healthcare systems. Taiwan is an attractive study setting to evaluate healthcare resource utilization and medical costs among RA patients in non-Western countries since the healthcare system of Taiwan is a fully publicly-funded single-payer universal care system and beneficiaries are unrestricted to go to hospitals or clinics of their choice, thereby excluding potential biases caused by variations of reimbursement schemes and insurance statuses in prior studies. In Taiwan, the prevalence of RA is 97.5/100,000, and middle-aged women are at especially high risk [9]. The aims of this study were to assess healthcare resource utilization and direct medical costs among RA patients receiving biologic therapies in Taiwan from a payer's perspective by utilizing a nationwide population-based claims database.

## Materials and methods

### Data source

The data used in this study were sourced from the National Health Insurance Research Database (NHIRD) in Taiwan, which covers 99% of the population of Taiwan of more than 23 million people. Data in the NHIRD that could be used to identify patients or care providers, including medical institutions and physicians, are scrambled cryptographically and then released in electronic format to the public annually for research purposes by the National Health Research Institute of Taiwan. Since the present study utilized de-identified secondary data, it was exempt from full review by The Joint Institutional Review Board of Taipei Medical University, Taiwan.

### Study population

The study population comprised RA patients (ICD-9-CM code: 714.0) who had received first-line tumor necrosis factor (TNF)-α antagonist treatment for at least 6 months between 2007 and 2011, were catastrophic illness cardholders for RA, and were aged 18 or older. After excluding patients with missing data, ultimately, 2425 patients were included in the analysis. The initial ambulatory care visit or hospitalization for receiving biologics of a RA patient was designated as the index date in the study. Furthermore, the baseline period was defined as the first 6 months after the index date of RA patients receiving first-line tumor necrosis factor (TNF)-α antagonist treatments of 48 injections of Etanercept (25 mg, twice a week) or 12 injections of Adalimumab (40 mg, once every 2 weeks) as regulated by the Bureau of National Health Insurance of Taiwan.

Those RA patients were further categorized into five subgroups: (1) single anti-TNF-α antagonist biologic treatment group-Etanercept (patients continued using the single biologic, Etanercept, after the baseline period); (2) single anti-TNF-α antagonist biologic treatment group-Adalimumab (patients continued using the single biologic, Adalimumab, after the baseline period); (3) multiple anti-TNF-α antagonist biologic treatment group-Switched (patients switched from one biologic agent to another and did not receive a second-line biologic treatment after the baseline period); (4) second-line biologic treatment group-Rituximab (patients received a second-line biologic treatment with Rituximab after the baseline period); and (5) only receiving biologics during the baseline period treatment group.

### Outcome measures

The outcome measures of the study were healthcare resource utilization and associated medical costs of the study cohort during the 1-year follow-up after the baseline period. Healthcare resource utilization and medical costs were further divided into RA-related and non-RA-related causes.

### Statistical analysis

Since medical costs data were heavily skewed to the right, the nonparametric method of Wilcoxon signed-rank test was performed to compare healthcare resource utilization and medical costs of those RA patients 1 year before the index date (the pre-RA period) and during the 1-year follow-up (the post-RA period), and were conducted for RA-related causes, non-RA-related causes, and all causes, separately.

All analyses were performed by using the SAS statistical package, version 9.3. A two-sided *P* value of less than .05 was considered statistically significant.

## Results

### Demographic characteristics and patterns of biologic therapies of patients

Table 1 lists demographic characteristics and patterns of biologic treatments of the study cohort. There were far more females than males in the study cohort, as expected. The mean age of those RA patients was around 55 years. In addition, the mean biologic costs during the 6-month baseline period were about two hundred and thirty thousand New Taiwan dollars (NT$), which corresponded to around US$7,807 (US$1 ≒ NT$29.46 in 2012).

**Table 1:** Demographic characteristics and patterns of biologic therapies of patients with rheumatoid arthritis (RA). (*N* = 2,425).

	Subgroups				
	Single TNF-α antagonist		Multiple biologics		Only 6 months of treatment (*N* = 69)
	Etanercept (*N* = 1,388)	Adalimumab (*N* = 738)	Switched (*N* = 57)	Second-line treatment (*N* = 173)	
*Sex*					
Female	1,141 (82.20%)	607 (82.25%)	50 (87.72%)	142 (82.08%)	55 (79.71%)
Male	247 (17.80%)	131 (17.75%)	7 (12.28%)	31 (17.92%)	14 (20.29%)
*Age (years)*, *mean (standard deviation)*					
	54.2 (12.81)	54.4 (12.43)	57.0 (11.94)	55.3 (12.76)	58.8 (12.19)
Maximum	89	86	77	93	82
Minimum	17	17	22	24	20
*Monthly insurance premium (NT$)*^a^					
≦ $15,840	555 (40%)	271 (37%)	24 (42%)	76 (44%)	34 (49%)
$15,841-$25,000	458 (33%)	243 (33%)	18 (33%)	49 (28%)	20 (29%)
$25,001-$35,000	168 (12%)	101 (14%)	7 (12%)	22 (13%)	6 (9%)
≧ $35,001	208 (15%)	123 (16%)	8 (13%)	26 (15%)	9 (13%)
*Biological costs during the 6-month baseline period*, *mean (NT$)*^a^					
	$229,790.74	$238,302.40	$228,430.26	$230,212.02	$216,508.23

^a^All nominal variables were deflated by the consumer price index. NT$ = New Taiwan Dollar. US$1 ≒ NT$29.46 in 2012.

With regard to patterns of biologic therapies, analytical results demonstrated that the most prescribed biologic agent for RA patients was Etanercept (*N* = 1,388; 57.2%). As a whole, 87.7% of RA patients received the same TNF-α antagonist (either Etanercept or Adalimumab) during the 1-year follow-up, 2.4% (*N* = 57) of those patients switched to another TNF-α antagonist after the baseline period, 7.1% (*N* = 173) of the study cohort received a second-line biologic agent (Rituximab), while the remaining patients discontinued use of any TNF-α antagonist.

### Differences in healthcare resource utilization and costs between the pre-RA and post-RA treatment periods

RA patients who received the single anti-TNF-α biologic treatment-Etanercept had more RA-related outpatient visits (means of pre-RA *vs*. post-RA: 19.9 *vs*. 21.9; *p* < 0.01), but shorter lengths of stay (1.3 *vs*. 1.1; *p* < 0.01) and fewer emergency room visits (0.8 *vs*. 0.5; *p* < 0.01). With respect to non-RA-related healthcare resource utilization, both outpatient visits (18.5 *vs*. 17.9; *p* < 0.05) and emergency room visits (1.2 *vs*. 1.1; *p* < 0.05) were reduced, but hospitalization days (0.3 *vs*. 0.4; *p* < 0.01) were increased after patients receiving Etanercept. As for total healthcare resource utilization, patients who were treated with Etanercept had more outpatient visits (38.4 *vs*. 39.8; *p* < 0.05), but shorter lengths of stay (1.7 *vs*. 1.4; *p* < 0.01) and fewer emergency room visits (2.0 *vs*. 1.6; *p* < 0.01) (Table 2).

**Table 2:** Differences in healthcare resource utilization and direct medical costs of patients with rheumatoid arthritis (RA) before and after the use of single biologic agent-Etanercept.

Single TNF-α antagonist	Pre-RA	Post-RA	Difference^a^
Etanercept			
*Healthcare resource utilization*, *mean*			
RA-related			
Number of outpatient visits	19.9	21.9	10.1%**
Hospitalization days	1.3	1.1	-15.4%**
Number of emergency room visits	0.8	0.5	-37.5%**
Non-RA-related			
Number of outpatient visits	18.5	17.9	-3.2%*
Hospitalization days	0.3	0.4	33.4%**
Number of emergency room visits	1.2	1.1	-8.3%*
RA + Non-RA			
Number of outpatient visits	38.4	39.8	3.6%*
Hospitalization days	1.7	1.4	-17.6%**
Number of emergency room visits	2.0	1.6	-20.0%**
*Medical costs (NT$)*^b^, *mean*			
RA-related			
Medication costs	$43,869.2	$367,465.2	737.6%**
Non-medication costs	$24,734.5	$24,810.2	0.3%
Total costs	$68,603.7	$392,275.4	471.8%**
Non-RA-related			
Medication costs	$4,480.0	$4,253.8	-5.1%*
Non-medication costs	$14,908.8	$14,651.7	-1.7%
Total costs	$19,388.8	$18,905.5	-2.5%*
RA + Non-RA			
Medication costs (biologics included)	$48,349.2	$371,719.0	668.8%**
Medication costs (biologics excluded)	$48,349.2	$35,867.0	-25.8%**
Non-medication costs	$39,643.3	$39,461.9	-0.5%
Total costs (biologics included)	$87,992.5	$411,180.9	367.3%**
Total costs (biologics excluded)	$87,992.5	$75,328.9	-14.4%**

^a^The Wilcoxon signed-rank test was used to test the difference between the pre-RA and post-RA periods.

^b^All nominal variables were deflated by the consumer price index. NT$ = New Taiwan Dollar. US$1 ≒ NT$29.46 in 2012.

* and ** represent significance at the 5% and 1% levels, respectively.

For RA-related medical expenditures, average medication costs increased noticeably after the treatment (pre-RA *vs*. post-RA: NT$43,869.2 *vs*. NT$367,465.2; *p* < 0.01), and so did total medical costs (NT$68,603.7 *vs*. NT$392,275.4; *p* < 0.01). By contrast, both non-RA-related medication costs (NT$4,480.0 *vs*. NT$4,253.8; *p* < 0.05) and total non-RA-related medical costs (NT$19,388.8 *vs*. NT$18,905.5; *p* < 0.05) were significantly lower after the treatment. The total medical costs (biologics included) were NT$411,180.9 during the 1-year follow-up. Still, exclusive of the costly biologic treatment, total medical costs were markedly reduced after those patients treated with Etanercept (NT$87,992.5 *vs*. NT$75,328.9; *p* < 0.01) (Table 2).

Table 3 displays the analytical results of differences in healthcare resource utilization and costs between the pre-RA and post-RA treatment time periods for patients receiving the biologic agent of Adalimumab. Similar to the results of Etanercept, patients who received Adalimumab had more RA-related outpatient visits (means of pre-RA *vs*. post-RA: 20.0 *vs*. 22.6; *p* < 0.01), but shorter lengths of stay (1.2 *vs*. 1.1; *p* < 0.05) and fewer emergency room visits (0.7 *vs*. 0.6; *p* < 0.01). The trend was also observed concerning total healthcare resource utilization.

**Table 3:** Differences in healthcare resource utilization and direct medical costs of patients with rheumatoid arthritis (RA) before and after the use of single biologic agent-Adalimumab.

Single TNF-α antagonist	Pre-RA	Post-RA	Difference^a^
Adalimumab			
*Healthcare resource utilization*, *mean*			
RA-related			
Number of outpatient visits	20.0	22.6	13.0%**
Hospitalization days	1.2	1.1	-8.3%*
Number of emergency room visits	0.7	0.6	-14.3%**
Non-RA-related			
Number of outpatient visits	17.7	18.0	1.7%
Hospitalization days	0.4	0.4	0.0%
Number of emergency room visits	1.0	0.9	-10.0%*
RA + Non-RA			
Number of outpatient visits	37.7	40.6	7.7%*
Hospitalization days	1.6	1.5	-6.3%*
Number of emergency room visits	1.7	1.5	-11.8%**
*Medical costs (NT$)*^b^, *mean*			
RA-related			
Medication costs	$43,014.1	$382,247.0	788.7%**
Non-medication costs	$38,123.7	$27,235.4	-28.6%**
Total costs	$81,137.8	$409,482.4	404.7%**
Non-RA-related			
Medication costs	$3,786.2	$3,543.5	-6.4%*
Non-medication costs	$14,180.9	$14,179.7	0.0%
Total costs	$17,967.1	$17,723.2	-1.4%
RA + Non-RA			
Medication costs (biologics included)	$46,800.3	$385,790.5	724.3%**
Medication costs (biologics excluded)	$46,800.3	$37,681.8	-19.5%**
Non-medication costs	$52,304.6	$41,415.1	-20.8%**
Total costs (biologics included)	$99,104.9	$427,205.6	331.1%**
Total costs (biologics excluded)	$99,104.9	$79,096.9	-20.2%**

^a^The Wilcoxon signed-rank test was used to test the difference between the pre-RA and post-RA periods.

^b^All nominal variables were deflated by the consumer price index. NT$ = New Taiwan Dollar. US$1 ≒ NT$29.46 in 2012.

* and ** represent significance at the 5% and 1% levels, respectively.

As for medical costs, RA-related medication costs (NT$43,041.1 *vs*. NT$382,247.0; *p* < 0.01) as well as total RA-related medical costs (NT$81,137.8 *vs*. NT$409,482.4; *p* < 0.01) increased prominently after patients receiving the biologic treatment. On the other hand, non-RA-related medication costs (NT$3,786.2 *vs*. NT$3,543.5; *p* < 0.05) were significantly lower in the post-RA treatment time period. Total medical costs (biologics included) of RA patients were NT$427,205.6, increasing noticeably during the 1-year follow-up. On the contrary, total medical costs (exclusive of biologics) were evidently lower after those patients went through the treatment (NT$99,104.9 *vs*. NT$79,096.9; *p* < 0.01) (Table 3).

With regard to those RA patients who switched from one biologic agent to another and did not receive a second-line biologic treatment after the baseline period, results indicated that there were significantly more RA-related outpatient visits (means of pre-RA *vs*. post-RA: 20.3 *vs*. 28.2; *p* < 0.01), longer lengths of stay (1.3 *vs*. 1.4; *p* < 0.05), and more emergency room visits (0.5 *vs*. 0.7; *p* < 0.01) after those patients received the treatment. Similar results were also observed regarding total healthcare resource utilization (Table 4).

**Table 4:** Differences in healthcare resource utilization and direct medical costs of patients with rheumatoid arthritis (RA) before and after the use of multiple biologic agents-Switched.

Multiple biologic agents	Pre-RA	Post-RA	Difference^a^
Switched			
*Healthcare resource utilization*, *mean*			
RA-related			
Number of outpatient visits	20.3	28.2	38.9%**
Hospitalization days	1.3	1.4	7.7%*
Number of emergency room visits	0.5	0.7	40.0%**
Non-RA-related			
Number of outpatient visits	20.0	22.2	11.0%**
Hospitalization days	0.1	0.1	0.0%
Number of emergency room visits	1.1	1.0	-9.1%*
RA + Non-RA			
Number of outpatient visits	40.3	50.4	25.1%**
Hospitalization days	1.4	1.5	7.1%*
Number of emergency room visits	1.6	1.7	6.3%*
*Medical costs (NT$)*^b^, *mean*			
RA-related			
Medication costs	$55,157.6	$353,396.5	540.7%**
Non-medication costs	$43,582.0	$44,963.7	3.2%
Total costs	$98,739.6	$398,360.2	303.4%**
Non-RA-related			
Medication costs	$2,719.3	$3,746.0	37.8%**
Non-medication costs	$13,976.0	$12,952.2	-7.3%**
Total costs	$16,695.3	$16,698.2	0.0%
RA + Non-RA			
Medication costs (biologics included)	$57,876.9	$357,142.5	517.1%**
Medication costs (biologics excluded)	$57,876.9	$58,339.6	0.8%
Non-medication costs	$57,558.0	$57,915.9	0.6%
Total costs (biologics included)	$115,434.9	$415,058.4	259.6%**
Total costs (biologics excluded)	$115,434.9	$116,255.5	0.7%

^a^The Wilcoxon signed-rank test was used to test the difference between the pre-RA and post-RA periods.

^b^All nominal variables were deflated by the consumer price index. NT$ = New Taiwan Dollar. US$1 ≒ NT$29.46 in 2012.

* and ** represent significance at the 5% and 1% levels, respectively.

On the subject of medical costs, RA-related medication costs (NT$55,157.6 *vs*. NT$353,396.5; *p* < 0.01) and total costs (NT$100,121.3 *vs*. NT$396,978.5; *p* < 0.01) both exhibited a substantial increase in the post-RA time period. Total medical costs (biologics included) of RA patients were NT$412,676.7 during the 1-year follow-up. In the same way, total medical costs (exclusive of biologics) were considerably lower after those patients embarked on the treatment (NT$115,434.9 *vs*. NT$101,873.8; *p* < 0.01) (Table 4).

As presented in Table 5, for those RA patients who received the second line biologic-Rituximab, they utilized more RA-related outpatient care (means of pre-RA *vs*. post-RA: 21.1 *vs*. 26.3; *p* < 0.01), more inpatient care (1.3 *vs*. 1.6; *p* < 0.01), as well as more emergency room service (0.7 *vs*. 0.9; *p* < 0.01). For overall healthcare resource utilization, there was a substantial increase of outpatient service (40.9 *vs*. 44.7; *p* < 0.01) and inpatient care (1.5 *vs*. 1.7; *p* < 0.01), but a reduction of emergency room service (2.0 *vs*. 1.9; *p* < 0.05) in the post-RA time period.

**Table 5:** Differences in healthcare resource utilization and direct medical costs of patients with rheumatoid arthritis (RA) before and after the use of second-line biologic agent-Rituximab.

Multiple biologic agents	Pre-RA	Post-RA	Difference^a^
Second-line treatment: Rituximab			
*Healthcare resource utilization*, *mean*			
RA-related			
Number of outpatient visits	21.1	26.3	24.6%**
Hospitalization days	1.3	1.6	23.1%**
Number of emergency room visits	0.7	0.9	28.6%**
Non-RA-related			
Number of outpatient visits	19.8	18.4	-7.1%*
Hospitalization days	0.2	0.1	-50.0%**
Number of emergency room visits	1.3	1.0	-23.1%**
RA + Non-RA			
Number of outpatient visits	40.9	44.7	9.3%**
Hospitalization days	1.5	1.7	13.3%**
Number of emergency room visits	2.0	1.9	-5.0%*
*Medical costs (NT$)*^b^, *mean*			
RA-related			
Medication costs	$44,345.4	$370,272.6	735.0%**
Non-medication costs	$48,201.2	$51,122.4	6.1%*
Total costs	$92,546.6	$421,395.0	355.3%**
Non-RA-related			
Medication costs	$6,032.9	$6,681.7	10.8%**
Non-medication costs	$16,228.0	$15,060.7	-7.2%*
Total costs	$22,260.9	$21,742.4	-2.3%
RA + Non-RA			
Medication costs (biologics included)	$50,378.3	$376,954.3	648.2%**
Medication costs (biologics excluded)	$50,378.3	$50,554.5	0.3%
Non-medication costs	$64,429.2	$66,183.1	2.7%
Total costs (biologics included)	$114,807.5	$443,137.4	286.0%**
Total costs (biologics excluded)	$114,807.5	$116,737.6	1.7%

^a^The Wilcoxon signed-rank test was used to test the difference between the pre-RA and post-RA periods.

^b^All nominal variables were deflated by the consumer price index. NT$ = New Taiwan Dollar. US$1 ≒ NT$29.46 in 2012.

* and ** represent significance at the 5% and 1% levels, respectively.

For both RA-related medication costs (NT$44,354.4 *vs*. NT$370,272.6; *p* < 0.01) and total medical costs (NT$95,467.8 *vs*. NT$418,473.8; *p* < 0.01), those expenditures increased considerably. As for total medical costs (biologics included) of RA patients undertaking Rituximab, they were NT$443,137.4 during the 1-year follow-up. Lastly, total medical costs (exclusive of biologics) of those patients were slightly higher in the post-RA treatment time period (NT$114,807.5 *vs*. NT$116,737.6; statistically insignificant) (Table 5).

Lastly, results of subgroup analysis all showed statistically insignificant cost ratios between female and male patients concerning direct medical costs (including biologics) during the 1-year follow-up, although male patients incurred slightly higher medical costs than their counterparts (cost ratios of male versus female—Etanercept: 1.014, *p* = 0.721; Adalimumab: 1.073, *p* = 0.144; Switched: 1.021, *p* = 0.683; Rituximab: 1.092, *p* = 0.095).

## Discussion

The present mirror-image study assessed healthcare resource utilization and direct medical costs among 2,425 RA patients receiving biologic therapies by using a national claims database in Taiwan from a payer's perspective. In general, analytical results revealed that the most prescribed TNF-α antagonist for RA patients was Etanercept (57.2%), and the majority of RA patients (87.7%) received the same TNF-α antagonist during the 1-year follow-up. Moreover, compared to 1 year before the index date, there were significant reductions in emergency room visits and hospitalization days for RA patients treated with the same anti-TNF-α biologic treatment during the 1-year follow-up. By contrast, those patients consumed more outpatient resources after undertaking biologic therapies. As for those RA patients who switched to another anti-TNF-α biologic treatment or received a second-line biologic agent, they utilized more outpatient and emergency room services as well as longer lengths of stay.

With regard to medical costs, this study demonstrated that the corresponding medication costs went up noticeably during the 1-year follow-up, mainly due to costly biologic therapies. On the other hand, nearly all total direct medical costs (biologics excluded) were significantly reduced across the study cohort after those RA patients went through biologic therapies.

In the current study, initiation of the most prescribed TNF-α antagonist-Etanercept led to significantly higher numbers of RA-related outpatient visits, but lower numbers of emergency room visits and hospitalization days among RA patients. The findings are in agreement with those from prior research [10]. It has been suggested that RA patients consuming more outpatient resources after undertaking biologic treatment may reflect close monitoring of those patients after initiating a new therapy [10].

Pertaining to medical costs, the results of the investigation are compatible with previous research. A previous national claims database study in Korea by Kwon and colleagues that found that medication costs were a leading cost driver of total medical costs of RA patients, and biologic treatment was a primary determinant of medical costs [11]. Similarly, another study done in France by Juillard-Condat and colleagues reported that after 1 month of using an anti-TNF-α antagonist, the average cost per patient with RA in the RA-related costs grew by 2.8-fold, and the medication costs per capita soared by 69.7% after 1 month of treatment[12]. The current analysis exhibited similar patterns of medical expenditures. Moreover, we also observed that there was a marked reduction in nearly all total direct medical costs (biologics excluded) across the study cohort during the 1-year follow-up. The lesser medical costs could be reasonably explained by the established fact that adherence to biologic treatment is associated with a reduction in overall medical costs among RA patients [13]. Furthermore, results of subgroup analysis demonstrated that male patients incurred slightly higher medical costs than their counterparts, albeit in a statistically insignificant fashion. These findings corroborate those from previous research [14].

As for those RA patients who switched to another TNF-α antagonist or received a second-line biologic agent, they had more outpatient visits, stayed in hospitals longer, and consumed more emergency room services during the 1-year follow-up. Along the same lines, results demonstrated that patients with RA who switched to a second first-line biologic therapy or received a second-line biologic agent incurred considerably higher medical costs, compared to those who continued treatment with their initial biologic agents. Findings of this study conform to the argument in the literature that that switching biologic therapy is associated with increased medical costs [15–18], and results in an effect size that is usually lower than that of a first biologic agent [19]. For instance, a previous investigation by Rosenblatt and colleagues revealed that monthly medical costs were 27% higher for patients who switched first-line biologic agents than those who did not switch [15].

The present study makes a significant contribution to the growing body of literature investigating healthcare resource utilization and medical costs among RA patients receiving biologic therapies. The main strength of the study is that as we take advantage of a population-based registry database, findings of this study likely represent the real-world evidence. Another asset of the research is the study of an Asian population; therefore, research findings of this investigation add to the literature where previous studies focused mostly on Western countries.

Limitations of this study are as follows. First, the analyses were conducted up to the time period of data availability for the present study, from 2007 to 2012. During the study period only Etanercept, Adalimumab, and Rituximab were covered by the NHI in Taiwan. Consequently, this investigation could only analyze related data pertaining to the three biologic agents. Second, we could only analyze direct medical costs extracted from the database, as information concerning out-of-pocket healthcare expenditures and productivity loss is not available in the database. Third, this research employed an observational cohort study design, as RA patients could not be randomly allocated to different treatment groups since we utilized a secondary database. As a result, the risk of selection bias remains a possibility. Lastly, information concerning disease severity of patients with RA (for example, disease activity score by 28 joints; DAS28) was not available in the NHIRD.

Rheumatoid arthritis is an irreversible chronic disease, and thus it constitutes a substantial burden on health care systems and societies due to treatment costs and productivity losses. The present study presented evidence that in the real-world management of RA, the great majority of patients had continuous treatment with no change of their index biologics. RA patients treated with biologics utilized fewer emergency room visits and shorter hospitalization days during follow-up, but incurred higher total medical costs, which might be due to increased utilization of outpatient services and associated biologic drug costs. Taken together, this study provides meaningful information on healthcare resource utilization and medical costs of RA patients for healthcare providers and policymakers.
